# ‘This Is Not Life’: Psychosocial Experiences of Hungarian Secondary School Students During COVID-19 School Closures: A Retrospective Qualitative Study

**DOI:** 10.3390/children13091180

**Published:** 2026-09-02

**Authors:** Gábor Pál Stromájer, Melinda Csima, Bernadett Varga, Tímea Stromájer-Rácz

**Affiliations:** 1 Institute of Basics of Health Sciences, Midwifery and Health Visiting, Faculty of Health Sciences, University of Pécs, H-7621 Pécs, Hungary; stromajer.gabor.pal@uni-mate.hu (G.P.S.); bernadett.varga@etk.pte.hu (B.V.); 2 Institute of Education, Hungarian University of Agriculture and Life Sciences, H-7400 Kaposvár, Hungary; 3 Institute of Diagnostic, Faculty of Health Sciences, University of Pécs, H-7621 Pécs, Hungary; timea.stromajer-racz@etk.pte.hu

**Keywords:** adolescents, COVID-19, school closures, online education, adolescent mental health, peer connectedness, social isolation, psychological distress, early prevention, psychosocial development

## Abstract

**Highlights:**

**What are the main findings?**

- Hungarian secondary school students described COVID-19 school closures not only as an educational disruption but also as a broader interruption of adolescent life, including peer relationships, school belonging, daily routines, and age-specific experiences.
- Recurring psychosocial burdens were related to social isolation, reduced peer contact, loss of school-based community experiences, uncertainty about graduation and the future, and the perceived disruption of normal adolescent life.

**What are the implications of the main findings?**

- During future pandemics or other prolonged crises, adolescent mental health support should go beyond maintaining digital education and should actively preserve peer connectedness, school belonging, daily structure, and emotional validation.
- Preventive and supportive interventions should be developmentally sensitive, addressing different needs among younger students, who may struggle with class community formation, and final-year students, who may be affected by uncertainty, transition rituals, and future orientation.

**Abstract:**

**Background/Objectives:** School closures and social restrictions during the COVID-19 pandemic substantially disrupted adolescents’ everyday lives. However, much of the existing evidence relies on standardized measures or retrospective accounts, providing less insight into how adolescents themselves described the psychosocial meaning of this disruption while it was still unfolding. This study aimed to explore Hungarian secondary school students’ real-time written reflections on their experiences during COVID-19-related school closures, online education, and social restrictions, with a focus on adolescent mental health, peer connectedness, school belonging, and developmental disruption. **Methods:** A retrospective qualitative study was conducted using 111 anonymized open-ended student reflections written in 2021 during the active period of online education and social restrictions. The texts were originally produced in a pedagogical-reflective context, not as research interviews or questionnaire responses. The material was analysed using structured qualitative content analysis. Two researchers independently coded the texts; disagreements were resolved through discussion, and a third researcher was involved when consensus was required. Descriptive frequency summaries were used to contextualize the qualitative findings. **Results:** In the descriptive frequency summaries, negative appraisals were most common in relation to the general pandemic situation, online schooling, and peer relationships. Thematic analysis identified recurring patterns of confinement, loss of normality, reduced peer contact, weakened school routines, academic overload, uncertainty about graduation and the future, and the loss of age-specific adolescent experiences and transition rituals. Family appeared as both a stabilizing resource and, in some cases, a source of conflict or additional burden. A central interpretive theme was that adolescents experienced the pandemic not merely as educational disruption but as an interruption of adolescence itself. **Conclusions:** The findings highlight that adolescent mental health support during large-scale crises should extend beyond maintaining educational continuity. Early prevention and intervention strategies should preserve peer connectedness, school belonging, daily structure, emotional validation, and developmentally sensitive support during future pandemics or comparable periods of prolonged social restriction.

## 1. Introduction

The SARS-CoV-2 virus, which emerged at the end of 2019, rapidly developed into a global pandemic that fundamentally reshaped everyday social life and affected people’s mental well-being [1]. To control the spread of COVID-19, non-pharmaceutical public health measures were widely implemented worldwide, including lockdowns, social distancing, quarantine, and the partial or complete closure of educational institutions [2]. Although these measures were necessary from an epidemiological perspective, they also imposed substantial psychosocial burdens, particularly on children and adolescents [3,4]. In Hungary, the first nationwide transition to out-of-classroom digital education came into effect on 16 March 2020. During subsequent pandemic waves, secondary school students again faced periods of online education and social restrictions. As a result, adolescents were placed for a prolonged period in a situation in which many of their usual social contacts, daily routines, and shared community experiences were substantially reduced or disappeared.

COVID-19 should not be interpreted solely as a single historical crisis. Zoonotic infectious diseases continue to represent a major global health risk, and the emergence of future pandemic situations cannot be excluded [5,6]. Therefore, examining the psychosocial consequences of the COVID-19 pandemic is important not only for understanding the recent past but also for preparing for future crises that may similarly affect young people’s everyday lives and mental health.

Adolescence is one of the most sensitive periods of development, during which identity formation, the development of autonomy, emotional regulation, and peer relationships become particularly important [7,8]. Friendships during this life stage do not merely fulfil a social function; they are fundamental contexts for self-esteem, emotional security, social learning, and coping with stress [9,10]. Social isolation during adolescence may therefore mean more than a reduction in interpersonal contact: it may also affect developmental processes related to autonomy, self-understanding, emotional security, future orientation, and identity formation. Studies conducted during the pandemic consistently reported increases in anxiety and depressive symptoms, loneliness, emotional exhaustion, loss of motivation, and uncertainty about the future among children and adolescents [11,12,13,14].

School is not only a place of learning during adolescence but also a key environment for psychosocial development [15]. Secondary school communities provide daily structure, social feedback, peer relationships, emotional support, and a sense of normality [16]. Online education therefore represented not only an organizational challenge for learning but also a restriction of the social and developmental experiences associated with school life. Several authors have emphasized that social deprivation and the loss of the school environment may have particularly strong effects on adolescent development and mental well-being [3,17].

Most studies examining the impact of COVID-19 on children and adolescents have used questionnaire-based, standardized, or quantitative methods [11,12]. These studies have provided important epidemiological data, but they are less able to capture how young people interpreted isolation, routine disruption, school closures, or the loss of peer contact from their own perspective. Although qualitative studies have explored adolescents’ experiences during COVID-19 [18,19,20], this specific type of real-time, naturally written student material remains underrepresented. The present study therefore offers a distinctive contribution by analysing anonymized student reflections that were produced in a pedagogical-reflective context during the active period of restrictions and were later examined through retrospective secondary analysis.

A narrative approach makes it possible to examine how participants express their experiences through their own meaning systems [21,22]. Such texts can provide direct insight into the emotional and social states experienced at the time, especially when they were produced while the restrictions were still ongoing rather than as later retrospective narratives [23].

The aim of this study was to explore the psychosocial experiences of Hungarian secondary school students during the COVID-19 pandemic through the analysis of open-ended, spontaneous student reflections.

## 2. Materials and Methods

### 2.1. Study Design and Data Source

This study was conducted as a retrospective, exploratory qualitative study aimed at examining the psychosocial experiences of Hungarian secondary school students during the COVID-19 pandemic. The analysis was based on previously produced, anonymized student essays that were examined through retrospective secondary analysis. The aim was to understand how young people described, in their own words, their experiences of social isolation, online education, reduced peer contact, disruption of daily routines, uncertainty about the future, and the interruption of age-specific adolescent life situations.

The analysed texts were produced during the 2020/2021 school year, in the period of online education and social restrictions associated with the COVID-19 pandemic, within a pedagogical-reflective context. A secondary school teacher invited students to write voluntary, anonymous, open-ended reflections on how the current situation was affecting them, how they were managing their school assignments, what they were doing in their free time, and how they were feeling in general. The prompt explicitly stated that responding was not compulsory and that responses should be submitted without names, anonymously. The full original Hungarian wording of the writing task and its English translation are provided in Supplementary File S1. The task was not designed as research data collection, a standardized questionnaire, or an interview, but as a pedagogical, self-reflective writing assignment. Students were able to describe their experiences in their own language, without predefined response categories.

A total of 111 essays written by Hungarian secondary school students were included in the present study. The documents were produced by students in Grades 9–12 who were enrolled in vocational secondary and vocational education settings. The students were between 14 and 19 years of age, and both boys and girls were represented in the sample (Table 1).

The final sample included 64 boys (57.7%) and 47 girls (42.3%). Students were enrolled in Grades 9–12: 47 students were in Grade 9, 21 in Grade 10, 19 in Grade 11, and 24 in Grade 12. The age range of the participants was 14–19 years. Individual-level age data were not available in the anonymized dataset; therefore, age could only be reported as a range. Because the essays were originally submitted anonymously and were not collected as part of a planned research study, only the available anonymized demographic variables could be used in the retrospective analysis.

The sample formation process was reconstructed retrospectively. Because the texts were originally produced as a voluntary pedagogical-reflective writing task rather than as part of a planned research study, the exact number of students who received the task could not be determined with certainty. The research team received all anonymous written responses that had been submitted in response to the task. A total of 111 essays were available for retrospective secondary analysis, and all 111 were included in the final analytic sample. No essays were excluded on the basis of length, level of detail, or depth of self-disclosure. Very brief responses were also retained in order to preserve the integrity of the available textual material and to avoid selective exclusion based on the richness of the response.

An important methodological feature of the data source is that the essays were not retrospective recollections produced after the pandemic period but were written during the active phase of restrictions, online education, and reduced opportunities for in-person social contact. This made it possible to examine how adolescents interpreted the emotional, social, and developmental experiences associated with the pandemic from within their current life situation.

### 2.2. Analysis of Student Essays

The open-ended texts were analysed using an exploratory qualitative approach based on structured qualitative content analysis. This method was considered appropriate for systematically identifying recurring themes, meanings, emotional emphases, and social experiences in student essays that varied in length and depth, while organizing the material through a transparent category system [24,25,26,27].

As a first step, members of the research team read the full textual material several times to become familiar with the data. An initial thematic category system was then developed inductively. The categories emerged primarily from the students’ own wording and from recurring content elements within the texts. During inductive category development, we also drew on the principles of thematic analysis, particularly the requirement that categories should be grounded in recurrent meanings identified in the textual material [28].

During coding, the complete essay was treated as the primary unit of analysis; however, longer responses or texts addressing multiple topics were also divided into smaller meaning units. Accordingly, a single student essay could be associated with more than one thematic category if it contained several distinct psychosocial contents. Each meaning unit, however, was consistently assigned to one main category.

After the preliminary category system had been developed, two researchers (GPS and MCS) independently coded the student essays. Independent coding was used to assess the consistent application of the categories and to reduce the possibility of bias arising from individual interpretation. In cases where the two coders assigned different categories, the disputed text segments were reviewed jointly. If the classification remained unclear, additional researchers (TSR and BV) were involved, and a consensus decision was reached.

Coding was conducted manually, and the coding results were recorded and managed in Microsoft Excel. No dedicated qualitative data analysis software was used. Manual coding was chosen because the student essays varied considerably in wording, length, emotional tone, and narrative style; therefore, the full texts had to be read and interpreted in context rather than processed through automated or software-assisted coding procedures. The Excel coding matrix contained the main categories, coded meaning units, evaluative directions, and coder notes used during the comparison and consensus process.

In addition to the qualitative analysis, descriptive frequency summaries were prepared along the main evaluative dimensions. The purpose of these summaries was not to test statistical hypotheses, estimate population-level prevalence, or identify causal relationships, but to provide an overview of which evaluative directions appeared more frequently within the available textual material. Descriptive frequencies were calculated only for the four main evaluative dimensions and for selected subgroup summaries by gender and grade level. The more detailed psychosocial themes presented in Section 3.2 were analysed qualitatively and are therefore described as recurring themes rather than as prevalence estimates.

### 2.3. Ethical Considerations

No new data collection, participant recruitment, direct contact, or intervention was conducted as part of this study. The analysis was based exclusively on previously produced, anonymized textual documents. During data processing, no information that could identify the students, the school, teachers, family members, or any other individuals was used. All information that could have enabled the direct or indirect identification of the persons or institutions concerned was removed or generalized.

The original writing task was completed before the present research study was planned and was conducted as a voluntary, anonymous pedagogical-reflective classroom activity. Students were explicitly informed that responding was not compulsory and that responses should be submitted without names. No research participation, clinical assessment, intervention, or publication was involved at the time when the essays were written. For the present study, the research team received only anonymized textual material and did not have access to the students’ names or other directly identifying information. Because the essays were produced before the present study was planned and were not collected as research data, prospective informed consent for participation in the present study could not have been obtained at the time of writing. The requirement for individual student and parental informed consent for the retrospective secondary analysis of the anonymized material was therefore reviewed and waived by the Research Ethics Committee.

The results are presented in aggregated form and illustrated with anonymized quotations. When selecting quotations, care was taken to ensure that they represented the relevant thematic category while not containing any identifiable, sensitive, or potentially disadvantageous information about the students, the school, teachers, family members, or other individuals. Ethical approval for the study was granted by the Research Ethics Committee of the Institute of Education, Hungarian University of Agriculture and Life Sciences, approval number 4/2025-NI, on 8 December 2025.

## 3. Results

### 3.1. Main Dimensions and Categorization of Responses

A total of 111 essays written by secondary school students were included in the study. The final analytic sample consisted of all 111 anonymous student essays made available to the research team. No responses were excluded from the analysis. The texts were analysed along four main dimensions: general appraisal of the situation, family background, peer relationships/friends, and school situation/online education. In each dimension, classification was based not merely on whether the topic was mentioned but on the context and emotional tone in which it appeared in the text. Statements were classified as positive when the given dimension was described as a supportive, stabilizing, or favourable experience. Text segments were classified as negative when they expressed loss, absence, conflict, overload, anxiety, or another unfavourable experience. The neutral/not mentioned category included cases in which the topic did not appear as a meaningful content unit or was not associated with a clear emotional evaluation.

In the full sample, negative statements clearly dominated the general appraisal of the situation: 78 students (70.3%) described their overall experience of the pandemic period unfavourably. A similarly high proportion of negative responses was observed in the school situation/online education dimension, where 77 students (69.4%) reported unfavourable experiences. Negative evaluations also predominated in relation to peer relationships and friends: 62 students (55.9%) described the reduction or absence of social relationships. Family background showed a different pattern: in this dimension, most responses were neutral, or family did not appear as a central theme (Table 2).

The gender-stratified results are presented in Table 3. Among both boys and girls, negative appraisals were most frequent in the general appraisal of the situation, peer relationships/friends, and school situation/online education dimensions (Table 3).

Negative evaluations of the general situation were observed in 43 boys (67.2%) and 35 girls (74.5%). Negative evaluations of peer relationships/friends were identified in 34 boys (53.1%) and 28 girls (59.6%), while negative evaluations of school situation/online education were present in 44 boys (68.8%) and 33 girls (70.2%). Family background showed a different pattern in both groups, with most responses classified as neutral or not mentioned. Because the study was not designed to test gender differences statistically, these findings are presented descriptively.

Based on the grade-level analysis, negative general appraisals were observed across all grades, particularly among Grade 12 students. Negative evaluations of peer relationships/friends were proportionally more frequent in Grades 9–10, whereas unfavourable experiences related to the school situation and online education appeared frequently across all grades (Table 4).

### 3.2. Main Psychosocial Themes Emerging from the Student Essays

In addition to categorization, the thematic analysis of the texts identified several recurring psychosocial patterns. The themes reported in this section are presented as qualitative patterns identified in the textual material. They are not intended to represent statistically tested differences or prevalence estimates. The thematic analysis identified several recurring psychosocial themes in the students’ accounts: confinement and loss of normality, reduced peer relationships, burdens related to online education, uncertainty about the future and graduation, the loss of age-specific adolescent experiences and transition-related events, the dual role of family background, and coping strategies. Identity formation was not coded as a separate empirical theme; rather, it was used in the Discussion as a developmental interpretive framework for understanding how the reported experiences related to adolescence as a life stage.

Confinement and the narrowing of everyday life appeared repeatedly in the essays. Several students described the restrictions not simply as an inconvenience but as the loss of freedom, familiar daily rhythms, and normal life. The responses often referred to monotony, aimlessness, loss of motivation, and the feeling that everyday life had lost its natural structure. A Grade 12 student wrote: “They took away the one thing in a person’s life, namely freedom. […] Everyone is at home, wasting away, and people become worn down by being locked in. It feels like being in prison.” Others emphasized the disruption of daily routines and biological rhythms, the loss of vitality, or the desire for their former life to return.

The loss or reduction of peer relationships was also a central theme. Students repeatedly indicated that online communication could not replace face-to-face meetings, the class community, time spent with friends, or spontaneous shared experiences. One Grade 11 student summarized this as follows: “It is very frustrating that these are supposedly the best years of our lives, and we are just sitting at home and missing out on everything.” Among Grade 9 students, the interruption of class community formation appeared particularly strongly, as several students felt that their class could not become a real community because of the lack of in-person presence.

In the accounts of students in higher grades, especially final-year students, the loss of expected adolescent experiences and transition-related events appeared repeatedly. Students described the absence of graduation-related ceremonies, school-leaving events, class trips, banquets, shared school programmes, and the final school year as the loss of expected shared experiences associated with this stage of life. A Grade 12 student wrote: “I do not know what it is like to be a final-year student, and now I never will. I do not know what a school-leaving ceremony is like. […] I did not experience it, although I should have.” Another final-year student stated: “No school-leaving ceremony, no graduation, no banquet… The last year is supposed to be the best, but for us it is the worst. They took away one of the best parts of our lives.”

Online education was evaluated predominantly negatively. The most frequently mentioned problems included the lack of teacher explanation, too many assignments, unmanageable deadlines, difficulties in processing learning material independently, reduced motivation, and the physical and mental burden of extended screen time. One student wrote: “This online education has a very bad effect on my grades; I have no motivation, and I do not feel the weight of the fact that I should study.” Among final-year students, the burden of online education was often connected with fears about graduation and the future.

In Grade 12 responses, uncertainty related to final examinations, further education, work, and becoming an adult appeared particularly strongly. One student expressed several layers of fear: “I am afraid of the final exam, I am afraid of what will happen afterwards, I am afraid of becoming an adult, and I am afraid of being alone.” In this context, graduation appeared not only as an academic task but also as a central symbol of an uncertain future.

The role of family background was more nuanced. In several responses, family appeared as a source of emotional support or as a stabilizing environment. In other cases, however, prolonged time at home led to conflicts, caregiving responsibilities, or additional burdens. Some students reported illness in the family, bereavement, financial difficulties, or being confined together with siblings. This suggests that family did not appear uniformly as either a protective or a stressful factor but played different roles depending on the individual life situation.

Several essays also contained details indicating psychological and physical strain. Students wrote about anxiety, loneliness, sleep disturbances, loss of appetite, loss of motivation, physical fatigue, hopelessness, and emotional exhaustion. One Grade 12 student expressed the experience of emotional isolation in a particularly condensed form: “I am not alone, yet I am lonely.”

Alongside unfavourable experiences, coping strategies and protective factors also appeared. These included sports, time spent in nature, listening to music, caring for animals, online or in-person contact with friends, family support, and establishing a personal daily routine. Some responses also mentioned certain advantages of online education, such as a more flexible schedule or not having to commute; however, these positive elements usually appeared alongside the absence of personal relationships and the school community.

The overarching motif of the thematic findings was the experience of “not life”. A Grade 12 student described the situation as the loss of freedom and normal life: “This is not a state of being, and it is not freedom, but most of all, it is not life.” Another final-year student similarly emphasized the loss of youth and social life: “Our youth has been completely taken away from us; we are locked in. I am not talking about parties here, but about our social life.” Overall, the responses showed that, for students, the pandemic period did not merely mean a change in education but also the experience of being unable to live through the natural social and developmental experiences of their age.

## 4. Discussion

The aim of the present study was to explore the psychosocial experiences of Hungarian secondary school adolescents during the COVID-19 pandemic through the analysis of spontaneous student essays produced during the active period of restrictions. Based on our findings, pandemic-related lockdowns and school closures were described by adolescents not merely as educational difficulties or temporary lifestyle changes but as an experience of disruption to normal adolescent life. The texts repeatedly referred to reduced peer relationships, the absence of the school environment, disruption of daily routines, uncertainty about the future, and the loss of age-specific transitions and shared community experiences.

One of the most important findings of the study is that the dominant psychosocial burden described by students was not related solely to changes in education but to the perceived disruption of the everyday social and developmental contexts of adolescence. These contexts included peer connectedness, school belonging, daily structure, shared experiences, coping with new demands, and future orientation. In this study, identity formation is therefore used as a developmental interpretive framework rather than as a directly coded empirical theme. This interpretation is consistent with developmental psychological perspectives that conceptualize adolescence as a sensitive period for autonomy, emotional regulation, peer relationships, self-understanding, identity formation, and preparation for adult roles [7,8]. Recent literature also emphasizes that the impact of COVID-19 was not limited to learning or individual indicators of mental health but also affected young people’s connectedness, sense of security, supportive environments, autonomy, and resilience [29].

### 4.1. The Pandemic as a Source of Social and Developmental Loss

Adolescence is characterized by increasing autonomy, intensified peer orientation, future planning, coping with new demands, and the gradual development of self-understanding and identity [7,23]. The student essays did not describe identity formation as an explicit theme; however, they repeatedly referred to several social and developmental contexts that are closely related to this process. Rather than describing only the educational challenges associated with school closures, participants emphasized the loss of shared experiences, the disruption of transition-related events associated with the final years of secondary school, and growing uncertainty surrounding graduation, further education, work, and adulthood.

The sense of loss was particularly visible in the accounts of final-year students. The cancellation of graduation-related ceremonies, school-leaving celebrations, the final school year as a shared experience, and other important milestones was described not only as the loss of individual events but also as the loss of expected shared experiences associated with this life stage. These occasions can be understood as symbolic markers of transition, school belonging, peer community, and future orientation. In this sense, they are relevant to the broader developmental context of adolescent identity formation, although they should not be interpreted as direct evidence of disrupted identity formation.

Previous qualitative and narrative studies conducted during the COVID-19 pandemic have similarly suggested that adolescents interpreted pandemic-related disruptions through processes of personal meaning-making and developmentally relevant coping. Velez et al. reported that adolescents sought to make sense of the profound changes affecting their school and social lives during the first year of the pandemic, linking these experiences to the ongoing process of identity development [30]. Likewise, the longitudinal study by Peplak et al. demonstrated that emotionally significant pandemic-related experiences became integrated into adolescents’ developing life narratives [31]. Our findings are consistent with this body of evidence while providing a distinctive perspective through the analysis of naturally occurring, real-time written reflections produced by Hungarian secondary school students during the active period of restrictions. Rather than retrospective recollections, these accounts captured how adolescents interpreted the disruption of their developmental trajectories while the crisis was still unfolding.

### 4.2. Peer Relationships and School as a Psychosocial Space

A recurring pattern in the student essays was the negative experience of reduced peer relationships. Based on the students’ accounts, online communication often could not replace in-person presence, spontaneous encounters, the everyday functioning of the class community, or shared adolescent experiences. Peer relationships should therefore not be interpreted merely as a matter of social convenience. During adolescence, peer connectedness is a fundamental context for self-esteem, autonomy, emotional regulation, social learning, and identity-related development [9,10]. Recent reviews also confirm that quarantine measures and social restrictions affected both objective and subjective indicators of adolescents’ peer relationships and were associated with increased loneliness and poorer mental health outcomes [32].

Descriptive grade-level patterns provide additional context for interpreting the findings. Negative evaluations of peer relationships/friends were proportionally more frequent in Grades 9–10 than in the higher grades, whereas negative appraisals of the general situation were most frequent among Grade 12 students. These descriptive patterns suggest that younger secondary school students may have been particularly affected by difficulties in class community formation and the strengthening of peer networks, while final-year students more often emphasized the loss of final-year experiences and uncertainty about graduation and the future. These patterns were not tested statistically and should therefore be interpreted as descriptive tendencies within the analysed material.

The absence of school was closely related to this pattern. In the students’ reflections, school appeared not only as a place of learning but also as a psychosocial space that provided daily structure, social feedback, a sense of community, in-person presence, and an experience of normality. This is consistent with developmental perspectives that conceptualize school as a key context for adolescent development [15]. The qualitative study by Widnall et al. (2022) similarly showed that school closures and lockdowns affected not only learning experiences but also daily routines, mental health, peer relationships, and uncertainty about returning to school [20]. Their longitudinal findings also indicated that lower levels of school and peer connectedness before the pandemic were associated with less favourable changes in mental health during the pandemic period [20,33].

### 4.3. Family Background and Psychological Strain

Family background showed a more nuanced pattern than the general appraisal of the situation, school, or peer relationships. Based on the categorized results, family remained neutral in most responses or did not appear as a primary source of stress. However, the more detailed analysis showed that its role was not uniform: for some students, family appeared as a supportive and stabilizing environment, whereas for others it was described as a source of conflict, caregiving responsibilities, or financial and health-related difficulties. This is consistent with reviews suggesting that family support, routine, parental warmth, activity, and problem-focused coping may have contributed to adolescents’ resilience, while family stressors and socioeconomic disadvantage may have increased vulnerability [29].

Several elements indicating heightened psychological strain also appeared in the texts, including anxiety, loneliness, sleep disturbances, loss of appetite, physical exhaustion, loss of motivation, hopelessness, and negative body-related experiences. These accounts are consistent with international studies reporting increased rates of anxiety and depressive symptoms among children and adolescents during the pandemic, as well as the adverse effects of loneliness and social isolation on mental health [2,11,12]. The systematic review by Viner et al. (2022) also identified unfavourable mental health patterns among young people during periods of school closures and social restrictions [34].

A distinctive feature of the present material is that these experiences did not appear as responses to standardized questionnaires but through the students’ own language. Expressions such as “I am not alone, yet I am lonely” simultaneously point to the difference between physical co-presence and emotional isolation and to the fact that being at home with others did not necessarily replace the peer connectedness that is important for adolescents. Psychological strain often emerged from the interaction of several factors: graduation, the future, academic performance, family burdens, the absence of social relationships, and the loss of age-specific experiences jointly shaped the experience of pandemic-related distress.

### 4.4. International Comparison and Concluding Interpretation

Our findings are consistent with previous qualitative studies examining adolescents’ experiences during COVID-19, including the study by Widnall et al. among adolescents in Southwest England and the analysis by Lukoševičiūtė and Šmigelskas among Lithuanian adolescents [20,35]. Similar to the British study, social isolation, the lack of in-person contact with friends, the loss of school routines, difficulties with online learning, uncertainty, and loss of motivation emerged as central themes in the present research.

At the same time, our study offers a distinctive contribution to the literature. First, it presents the experiences of Hungarian secondary school adolescents, a group that remains less represented in international qualitative research on COVID-19. Second, the analysed essays were not retrospective recollections produced after the pandemic period but naturally written student reflections created during the active phase of lockdowns and social restrictions. This made it possible to capture how young people interpreted the pandemic period from within their immediate life situation.

Overall, the findings suggest that, within this specific set of student essays, the psychosocial burden was not limited to online education, academic overload, or uncertainty related to the pandemic situation. Rather, students repeatedly described the disruption of everyday adolescent life, including restrictions on peer relationships, the absence of the school community, the loss of final-year experiences, and fears about the future. Together, these experiences shaped students’ accounts of the pandemic as a perceived disruption of normal adolescent life and of several social and developmental contexts relevant to adolescence. In this sense, the phrase that inspired the title—“This is not a state of being, and it is not freedom, but most of all, it is not life”—should not be interpreted as an isolated or extreme statement but as a condensed expression of the perceived loss of normality, connectedness, and age-specific experiences within the analysed texts.

### 4.5. Implications for Prevention and Support

These findings have implications for adolescent mental health prevention and support during future pandemics or other crises involving prolonged social restrictions. In such situations, support for adolescents should not be limited to maintaining educational processes, ensuring the technical conditions of digital education, or compensating for missed learning content. Preserving peer connectedness, maintaining the psychosocial role of the school community, supporting daily structure, and creating opportunities for emotional expression may also be important.

Preventive and supportive interventions should also take developmental differences into account. Among younger secondary school students, the disruption of class community formation and peer relationship development may be especially burdensome. Among final-year students, uncertainty about graduation, the future, becoming an adult, and the loss of transition-related events may become more prominent. Support should therefore be based not only on general mental health messages but also on the specific developmental needs of adolescents.

One practical implication of the study is that being heard and emotionally validated may itself have supportive value for adolescents. One student explicitly thanked the teacher for showing interest in students’ experiences, illustrating young people’s need for adult and institutional environments in which they can safely express their feelings. This insight extends beyond the specific context of COVID-19: in supporting adolescent psychosocial well-being, authentic adult attention, a sense of belonging, and recognition of age-specific developmental needs may be of fundamental importance.

## 5. Limitations

Several limitations should be considered when interpreting the findings of this study. First, the study was based on a non-representative sample; therefore, the results cannot be generalized to the entire population of Hungarian secondary school adolescents. The analysed essays originated from a specific school and social context, and the patterns presented primarily reflect the pandemic-related experiences of this particular student group.

Second, the study was based on the anonymized, retrospective secondary analysis of student essays that had been produced earlier in a pedagogical-reflective context. The texts were not created for research purposes and did not form part of a standardized interview or questionnaire but were written as open-ended, self-reflective assignments initiated by a teacher. This limits the comparability and uniformity of the responses; at the same time, it also represents one of the distinctive strengths of the study, as students described their pandemic-related experiences naturally and in their own language.

Third, although the original writing task was open-ended and did not explicitly require negative responses, it asked students to reflect on how the current pandemic situation was affecting them, how they were managing school assignments, and how they were feeling. Therefore, the possibility of a prompt framing effect cannot be excluded. The predominance of negative appraisals should consequently be interpreted as a pattern within this specific prompted, pedagogical-reflective textual material rather than as an unbiased estimate of adolescents’ overall pandemic experiences.

Fourth, the essays varied in length, level of detail, and depth of self-disclosure. Some students provided longer and emotionally richer accounts, whereas others responded briefly or descriptively, and some texts contained only limited analysable content. All available anonymous responses were nevertheless retained in the analysis, regardless of length or richness, in order to avoid selective exclusion of less detailed accounts. This may have influenced which themes became more prominent during the analysis.

Fifth, because no new data collection, participant contact, or follow-up was conducted, it was not possible to clarify or expand on the students’ statements or to explore their later life situations. The analysis was based exclusively on the available anonymized textual documents; therefore, the findings reflect subjective experiences expressed at a specific period of the pandemic.

Sixth, the frequency data are descriptive in nature and should not be interpreted as prevalence estimates. The proportions of categories were used to provide an overview of internal patterns within the textual material, rather than to test statistical hypotheses or draw causal conclusions.

Finally, although a structured coding process was applied, qualitative content analysis necessarily involves interpretive elements. The consistency of categorization was supported by independent coding by two researchers, discussion of discrepancies, and, when necessary, the involvement of additional researchers. Nevertheless, the findings should be understood as an interpretation of the psychosocial meanings expressed in the student essays.

## 6. Conclusions

Based on the findings of this study, school closures and social restrictions during COVID-19 were described by students as more than a change in education. In the student essays, the pandemic period appeared as a narrowing of social relationships, the loss of the school environment, the disruption of daily routines, growing uncertainty about the future, and an interruption of age-specific adolescent experiences. The psychosocial burden described in the essays was therefore not related solely to online education or to the pandemic situation itself but also to students’ perception that they were unable to live through the expected social, communal, and age-specific experiences of their life stage.

The study highlights the importance of understanding adolescent pandemic experiences not only through educational or clinical indicators but also through the social and developmental contexts in which adolescents normally live, learn, form relationships, and prepare for future transitions. These findings underline the need for developmentally sensitive approaches to adolescent support during future crises involving prolonged disruption of school life and peer connectedness.

## Figures and Tables

**Table 1:** Basic characteristics of the analytic sample.

Characteristic	n (%) or Range
Total sample	111
Boys	64 (57.7%)
Girls	47 (42.3%)
Grade 9	47 (42.3%)
Grade 10	21 (18.9%)
Grade 11	19 (17.1%)
Grade 12	24 (21.6%)
Age range	14–19 years

**Table 2:** Categorization of responses in the full sample across the four main dimensions.

Dimension	Positive n (%)	Neutral/Not Mentioned n (%)	Negative n (%)
General appraisal of the situation	12 (10.8%)	21 (18.9%)	78 (70.3%)
Family background	11 (9.9%)	85 (76.6%)	15 (13.5%)
Peer relationships/friends	9 (8.1%)	40 (36.0%)	62 (55.9%)
School situation/online education	7 (6.3%)	27 (24.3%)	77 (69.4%)

**Table 3:** Categorization of responses by gender across the four main dimensions.

Dimension	Boys Positive n (%)	Boys Neutral/Not Mentioned n (%)	Boys Negative n (%)	Girls Positive n (%)	Girls Neutral/ Not Mentioned n (%)	Girls Negative n (%)
General appraisal of the situation	5 (7.8%)	16 (25.0%)	43 (67.2%)	7 (14.9%)	5 (10.6%)	35 (74.5%)
Family background	4 (6.2%)	53 (82.8%)	7 (10.9%)	7 (14.9%)	32 (68.1%)	8 (17.0%)
Peer relationships/ friends	2 (3.1%)	28 (43.8%)	34 (53.1%)	7 (14.9%)	12 (25.5%)	28 (59.6%)
School situation/ online education	2 (3.1%)	18 (28.1%)	44 (68.8%)	3 (6.4%)	11 (23.4%)	33 (70.2%)

**Table 4:** Negative response patterns by grade level across the four main dimensions.

Grade Level	n	General Negative n (%)	Family Negative n (%)	Peer Relationships Negative n (%)	School/Online Education Negative n (%)
Grade 9	47	28 (59.6%)	8 (17.0%)	28 (59.6%)	33 (70.2%)
Grade 10	21	15 (71.4%)	2 (9.5%)	14 (66.7%)	14 (66.7%)
Grade 11	19	14 (73.7%)	2 (10.5%)	9 (47.4%)	15 (78.9%)
Grade 12	24	21 (87.5%)	3 (12.5%)	11 (45.8%)	15 (62.5%)

## Data Availability

The data supporting the findings of this study are not publicly available due to ethical and privacy restrictions. The study was based on anonymous student essays produced in a pedagogical-reflective context; however, the full textual dataset cannot be shared because the essays contain personal reflections and contextual details that may increase the risk of indirect identification, even after anonymization. Public sharing of the full textual material was not covered by the ethical approval. Relevant anonymized excerpts are included in the article.

## Supplementary Materials

The following supporting information can be downloaded at https://www.mdpi.com/article/10.3390/children13091180/s1: File S1: Original writing task.
