# Supplementary material for: ‘This Is Not Life’: Psychosocial Experiences of Hungarian Secondary School Students During COVID-19 School Closures: A Retrospective Qualitative Study
Source: Children (Basel). 2026 Sep 2;13(9):1180. doi: 10.3390/children13091180 (PMC13605980; doi:10.3390/children13091180)
Supplement: Supplementary file 1 [file children-13-01180-s001.zip › children-4518139-supplementary.pdf]

Supplementary File S1. Original Writing Task

Original Hungarian prompt

Sziasztok. Érdekes kihívással állunk szemben mindannyian manapság. Egy olyan helyzetben vagyunk, amivel eddig még nem találkoztunk, sem ti, sem mi tanárok. Így az online oktatás során csak az iskolai feladatokra koncentráltunk eddig, de engem érdekel, rátok milyen hatással van a jelenlegi helyzet. Kérlek, ha van kedvetek, akkor osszátok meg velem, hogyan boldogultok a feladataitokkal, mit csináltok a szabadidőben, hogy ne unatkozzatok, és egyáltalán hogyan érzitek magatokat. A válaszadás természetesen nem kötelező, de ha van mondanivalótok, akkor írjátok le. Név nélküli, anonim válaszokat várok.

English translation

Hello everyone. We are all facing an interesting challenge these days. We are in a situation that neither you nor we teachers have ever encountered before. So far, during online education, we have focused only on school assignments, but I am interested in how the current situation is affecting you. Please, if you feel like it, share with me how you are managing your assignments, what you are doing in your free time so that you do not get bored, and, in general, how you are feeling. Responding is, of course, not compulsory, but if you have something to say, please write it down. I am expecting responses without names, anonymously.
